# White matter tract microstructure and cognitive performance after transient ischemic attack

**DOI:** 10.1371/journal.pone.0239116

**Published:** 2020-10-23

**Authors:** Sana Tariq, Adrian Tsang, Meng Wang, Noaah Reaume, Helen Carlson, Tolulope T. Sajobi, Richard Stewart Longman, Eric E. Smith, Richard Frayne, Christopher D. d’Esterre, Shelagh B. Coutts, Philip A. Barber

**Affiliations:** 1 Seaman Family MR Center, Foothills Medical Centre, Calgary, AB, Canada; 2 Hotchkiss Brain Institute, University of Calgary, Room 1A10 Health Research Innovation Center, Calgary, AB, Canada; 3 Department of Community Health Sciences & O’Brien Institute for Public Health, University of Calgary, Calgary, AB, Canada; 4 Calgary Pediatric Stroke Program, Alberta Children’s Hospital Research Institute, Calgary, AB, Canada; 5 Alberta Health Services, Neuropsychology Service, Foothills Medical Centre, Calgary, AB, Canada; 6 Department of Clinical Neurosciences, Calgary Stroke Program, Foothills Medical Centre, Calgary, AB, Canada; 7 Department of Radiology, Foothills Medical Centre, Calgary, AB, Canada; Texas State University, UNITED STATES

## Abstract

**Background and purpose:**

Patients with transient ischemic attack (TIA) show evidence of cognitive impairment but the reason is not clear. Measurement of microstructural changes in white matter (WM) using diffusion tensor imaging (DTI) may be a useful outcome measure. We report WM changes using DTI and the relationship with neuropsychological performance in a cohort of transient ischemic attack (TIA) and non-TIA subjects.

**Methods:**

Ninety-five TIA subjects and 51 non-TIA subjects were assessed using DTI and neuropsychological batteries. Fractional anisotropy (FA) and mean diffusivity (MD) maps were generated and measurements were collected from WM tracts. Adjusted mixed effects regression modelled the relationship between groups and DTI metrics.

**Results:**

Transient ischemic attack subjects had a mean age of 67.9 ± 9.4 years, and non-TIA subjects had a mean age 64.9 ± 9.9 years. The TIA group exhibited higher MD values in the fornix (0.36 units, *P* < 0.001) and lower FA in the superior longitudinal fasciculus (SLF) (-0.29 units, *P* = 0.001), genu (-0.22 units, *P* = 0.016), and uncinate fasciculus (UF) (-0.26 units, *P* = 0.004). Compared to non-TIA subjects, subjects with TIA scored lower on the Addenbrooke’s Cognitive Assessment-Revised (median score 95 vs 91, *P =* 0.01) but showed no differences in scores on the Montreal Cognitive Assessment (median 27 vs 26) or the Mini-Mental State Examination (median 30). TIA subjects had lower scores in memory (median 44 vs 52, *P <* 0.01) and processing speed (median 45 vs 62, *P <* 0.01) but not executive function, when compared to non-TIA subjects. Lower FA and higher MD in the fornix, SLF, and UF were associated with poorer performance on tests of visual memory and executive function but not verbal memory. Lower FA in the UF and fornix were related to higher timed scores on the TMT-B (*P <* 0.01), and higher SLF MD was related to higher scores on TMT-B (*P <* 0.01), confirming worse executive performance in the TIA group.

**Conclusions:**

DTI scans may be useful for detecting microstructural disease in TIA subjects before cognitive symptoms develop. DTI parameters, white matter hyperintensities, and vascular risk factors underly some of the altered neuropsychological measures in TIA subjects.

## Introduction

Transient ischemic attack (TIA) is associated with cognitive impairment where more than 35% of subjects have impairment in multiple cognitive domains not explained by silent brain infarcts [1]. TIA is also associated with a four-fold increase in the risk of dementia in the first year following a TIA [2], but the cause of the progression from cognitive impairment to dementia is not well understood [3]. Dementia risk is probably related to the co-existence of vascular and neurodegenerative pathologies, the burden and location of acute vascular lesions, as well as, the complex interaction of vascular, lifestyle-related, and individual risk factors (age and education) [4]. Nonetheless, it is accepted that early disease in the brain likely develops decades before symptoms are detected, thereby providing an important opportunity to prevent or delay the onset of late-life cognitive decline.

In the absence of treatment for late onset cognitive decline, prevention provides the best option for avoiding or delaying the progression of cognitive impairment. The difficulty with detecting incipient disease before symptoms are present or are minimal relates to identifying broad pathological and clinical heterogeneity of disease [4]. Predicting underlying pathology clinically and radiologically can be challenging and especially so in the preclinical phase of disease. As such, the use of clinical neuroimaging techniques to assess the benefit of prevention strategies or disease-specific treatments is problematic. Neurodegenerative and vascular disease—common causes of late onset cognitive impairment—have long been linked to neural networks by the clinical and anatomical progression of disease in subjects [1, 2]. The existence of sensitive imaging techniques [5, 6], such as diffusion tensor imaging (DTI), has permitted the measurement of spatially orientated anatomical connections within these brain networks in vivo.

This current observational study aims to address these issues by exploring the relationship of white matter (WM) structures in subjects without symptoms of cognitive impairment presenting with TIA and non-TIA subjects with formal neuropsychological assessment. We sought (1) to determine that TIA subjects would exhibit WM microstructural differences in WM structures consistent with neurodegeneration or vascular damage compared to non-TIA subjects, and (2) to determine that these differences in diffusion measures would correlate with performance on neuropsychological tests. Establishing a relationship of DTI parameters in WM tracts with cognitive function would provide support to investigate the utility of DTI as a potential biomarker for clinical trials studying treatment interventions by comparing DTI measures of change with established MRI and neuropsychological measures.

## Methods

### Study population

Consecutive subjects presenting with first documented TIA, and non-TIA subjects (ages 45–75) were prospectively consented to the Predementia Neuroimaging of Transient Ischemic Attack (PREVENT) study [7] approved by the University of Calgary Research Ethics Board. Transient ischemic attack subjects were recruited through the stroke prevention clinic or the emergency department acutely (within 24 hours) and underwent an MRI scan and neuropsychological testing on the same day. Written informed consent was obtained from all subjects. Transient ischemic attack was defined according to the National Institute of Neurological Disease and Stroke criteria (NINDS, 1975). The study’s senior neurologist (PAB) formally reviewed and confirmed the TIA in all cases and subjects with persisting focal neurological symptoms in excess of 24 hours, or an alternative diagnosis was considered at 90-day clinical follow-up with subsequent exclusion. Consecutive non-TIA subjects were recruited from the community or spousal members were approached during the same period of recruitment as TIA subjects. Inclusion criteria for the PREVENT study of TIA subjects or non-TIA subjects included: a Mini-Mental State Examination (MMSE) [8] score between 24–30 (inclusive), absence of significant impairment in cognitive functions or activities of daily living; absence of dementia (Clinical Dementia Rating CDR 0–0.5); and no depression. TIA subjects or non-TIA subjects were excluded if there was: significant neurological disease or history of significant head trauma; psychiatric disorders, substance abuse or dependence within the past two years as defined by DSM-V criteria [9]; any significant systemic illness (cardiac failure, severe renal failure); current use of specific sedating medications; and contraindication to MRI.

### Clinical data collection

At study entry, baseline evaluation included clinical review, fasting cholesterol, glucose, and renal function. Detailed clinical history, including medications that could influence cognition were collected. We screened for obstructive sleep apnea using a standardised questionnaire [10]. Blood pressure (BP) measurement was recorded from the average of three sitting BPs at baseline and each subsequent visit. Vascular risk factors were recorded and computed into the Framingham Risk Score (FRS) [11] and included: smoking, diabetes, total cholesterol, HDL cholesterol, systolic blood pressure (BP), medication for BP, and family history of cardiovascular disease. The FRS are expressed as a numerical value with higher numbers reflecting higher vascular risk. All participants were managed according to current stroke prevention guidelines [12].

A stroke neurologist (PAB) reviewed all images. Acute infarct location was recorded as follows: (1) cortical (2) deep (basal ganglia, internal capsule, thalamus, and deep WM tracts) (3) cortical and deep, and (4) infratentorial. Diffusion weighted imaging (DWI) lesion volume was segmented and relative DWI intensity thresholds were calculated using MeVisLab software [13]. All PREVENT subject demographic, clinical, group disposition (TIA vs non-TIA subject) and imaging data was anonymized.

### Neuropsychological battery

PREVENT subjects underwent neuropsychological testing based on a standardized battery of tests closely aligned with that of the Alzheimer’s Disease Network Initiative (ADNI). Cognitive testing focused on the domains most affected by stroke, including attention/processing speed and executive function abilities [14], as well as, tests of verbal and visual memory. Three cognitive screens were employed: the Montreal Cognitive Assessment (MoCA) [15], Addenbrooke’s Cognitive Assessment-Revised (ACE-R) [21] and the MMSE [8] (built into ACE-R). Tests of memory included the Brief Visuospatial Memory Test-Revised (BVMT-R) [16], the memory component of (ACE-R) [17], and the WHO/UCLA Auditory Verbal Learning Test (AVLT) [18]. Tests of executive function included CLOX-1 and Trail Making Test B (TMT-B) [19]. Tests of processing speed included Digit Symbol (DS) Coding [20] and Trail Making Test A (TMT-A) [19]. Trail Making Test A and B were negatively scored where higher (time) scores denoted poor performance. The National Adult Reading Test’s (NART) [21] verbal IQ score was used as a surrogate of intellect. The domain scores were normalized for age, sex, and education using normative data provided with test manuals or previously published literature and calculated by averaging the T-scores from each of the individual tests.

### Image acquisition

All subjects underwent MR scans using a 3.0-T scanner (Discovery 750; General Electric Healthcare, Waukesha, Wisconsin, USA). T1-weighted (T1-w) anatomical images were acquired in the sagittal plane using a 3D inversion recovery prepared spoiled gradient-echo sequence (FOV = 25.6 cm; 180 1-mm slices; in-plane matrix size = 256 × 256; echo time (TE) ≈ 2.5 ms; repetition time (TR) ≈ 6.3 ms; inversion time (TI) = 650 ms; flip angle = 8°). T2-weighted (T2-w) fluid attenuated inversion recovery (FLAIR) images were acquired in the axial plane (FOV = 24 cm, 70 1-mm slices; acquisition matrix = 256 × 256; TE/TR/TI/flip = 140 ms/9000 ms/2250 ms/90°, echo train length (ETL) = 32). DTI sequences were collected using a single-shot spin echo-planar imaging (EPI) sequence (FOV = 24 cm; 3-mm slices; acquired matrix = 80 × 80 interpolated to 256 × 256; TR/TE = 70–80 ms/9,000–10,000 ms) with diffusion sensitizing gradients applied in 31 non-collinear directions (*b* = 1000 s/mm^2^) and with four *b* = 0 s/mm^2^ acquisitions.

### Image analysis

T1-w images were processed using FreeSurfer (using the recon-all option) to parcellate the brain according to a standard pipeline, and intracranial volume (ICV) was calculated using tools in the FMRIB Software Library (FSL, version 5.0.8; http://www.fmrib.ox.ac.uk/fsl) for the purposes of normalizing tract volumes to ICV. After processing, brain surface area for each participant was extracted. Diffusion tensor imaging (DTI) images were pre-processed using the standard FSL pipeline, including simple head motion and eddy current correction. The diffusion tensor was calculated using Diffusion Toolkit (http://trackvis.org/dtk/) and colour-coded FA and MD maps were generated. Data with poor co-registration, excessive motion, and/or incorrect tensor calculation (as observed by visual inspection) were removed. All images were anonymized, and image analysis was blind to demographic and clinical information.

### DTI tractography

**Fig 1:**
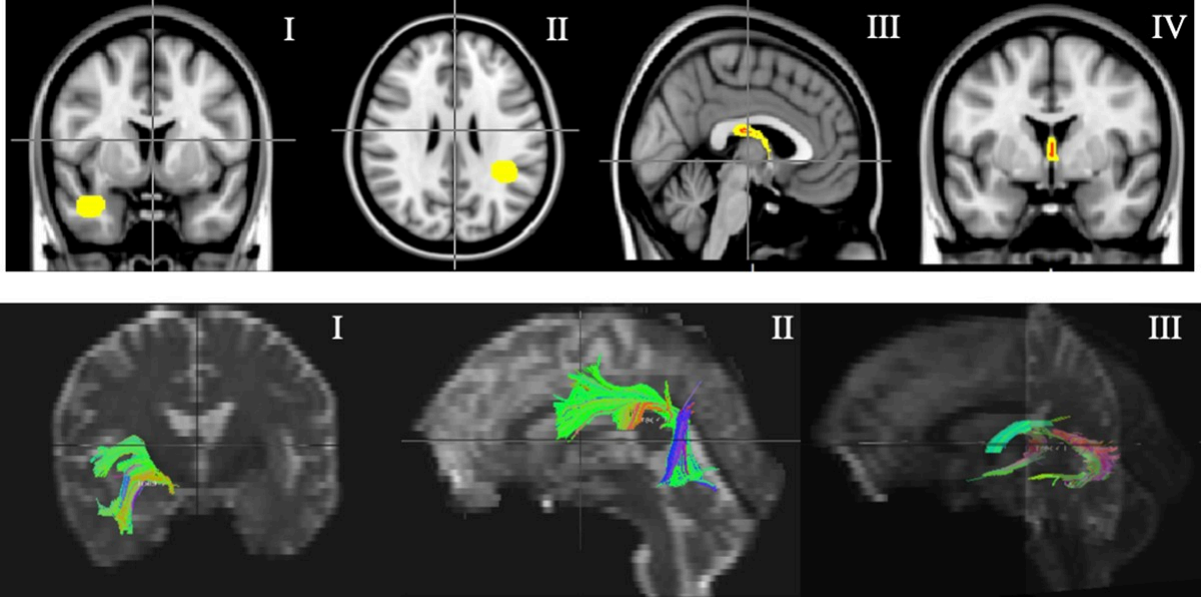
(A) Region-of-interest (ROI) placements for left uncinate fasciculus (UFL) (I), right superior longitudinal fasciculus (SLFR) (II) and fornix (III/IV). (B) Tract delineation for left uncinate fasciculus (UFL) (I), right superior longitudinal fasciculus (SLFR) (II) and fornix (III).

The T1-w images were normalized to MNI152 template space. Predetermined regions-of-interest (ROIs, Fig 1A) used to isolate tracts of interest were manually drawn on the template image using the JHU White Matter Tractography and Juelich Histological atlas available in FSL. Regions were placed on six tracts: cingulum gyrus (CG), parahippocampal cingulum (PHC), superior longitudinal fasciculus (SLF), uncinate fasciculus (UF), fornix, and the genu of the corpus callosum; left and right side where appropriate (Fig 1A) based on the implications for neuropsychological function i.e. relationship between tract and function. Regions were chosen based on highest fibre density and then registered from the template to imaging space of each subject using the FLIRT function in FSL.

TrackVis from the Diffusion Toolkit was used to delineate the tracts by deterministic tractography using the second order Runge-Kutta algorithm [22] with the FA threshold set to 0.20 to exclude gray matter, and the angle threshold set to < 35° (< 45° in the fornix) to exclude tracks with sharp curvature. Minimal manual correction of the tracts was done using the NOT operation sparingly to prune projections from neighbouring tracts; a diffusion tensor imaging atlas [23] was referenced to ensure anatomical accuracy. Sample tracts are illustrated in Fig 1B. Reconstructed tracts were then overlaid on FA and MD maps and the mean and standard deviation values were extracted for each using FSLMaths function. To ensure a fair comparison of diffusivity measures between subjects with variable brain size and to account variation in WM tract volume, intracranial volume (ICV) was calculated using tools in FSL and used as a covariate in the linear mixed effects model.

### White matter hyperintensity volume

Cerebra-WML software was used to measure the volume (in mL) of WM hyperintensities on the T2-w FLAIR-images [24], following international recommendations for the reporting, image acquisition, and analysis of small vessel disease reported by our group [24]. Processing steps involve skull stripping, cerebrospinal fluid (CSF) removal, and subsequent segmentation of WM. This semi-automated method allows for low subjectivity and high standardization, reproducibility and traceability [25, 26].

### Statistical tests

The statistical analyses were conducted in SAS (v9.4 SAS Institute Inc., 2013). Sample characteristics were described using mean, standard deviation, median, and percentile values as well as frequency distributions. Differences between the TIA and non-TIA groups were compared using independent *t*-test or Wilcoxon rank-sum test and Chi-squared or Fisher’s exact tests. For all the regressions, we standardized the continuous variables including all the DTI-derived FA and MD measures, age, ICV, WM hyperintensities volume, premorbid intellect, and DWI volume. In order to test if TIA subjects exhibit changes in DTI metrics compared to the non-TIA group, linear mixed effect (LME) models were used to assess the DTI-derived FA and MD measures. Specifically, fixed effects included group (TIA vs. non-TIA), WM tract (CG, PHC, fornix, SLF, UF, and genu), TIA by WM tract interaction, age, FRS, ICV, WM hyperintensities volume, and premorbid intellect. Random effects include subjects, and hemisphere (nested within different tracts). In the LME models, our primary predictors of interest were group, WM tract, and TIA by WM tract interaction. Age, FRS, ICV, WM hyperintensities volume, and premorbid intellect were adjusted for in the models and any two-way modifications were tested. In order to explore the FA and MD WM tract measures association with verbal and visual memory, and executive function cognitive domain measures, multiple linear regressions were performed. Important covariates were adjusted, and any two-way modifications were tested. Since multiple linear regression were conducted, the *P* values were adjusted based on false discovery rate [27, 28]. All results were considered statistically significant with a two-sided *P*-value of less than 0.05.

## Results

### Demographics

In total, there were 146 PREVENT subjects: 95 TIA subjects (50 male and 45 females; mean age of 67.9 ± 9.4 years), and 51 non-TIA subjects (17 males and 34 females; mean age 64.9 ± 9.9 years). Twenty-five subjects presenting with TIA (26%) were detected to have small DWI lesions (mean volume 0.63 ± 0.87 ml); DWI lesions were mainly cortical (19/25, 76%) except for three DWI lesions in the deep WM (12%), two in the thalamus (8%) and one in the pons (4%). The demographic, clinical, and cognitive screening results are summarized in Table 1. Compared to non-TIA subjects, TIA subjects scored lower on the ACE-R (median score 95 vs 91, *P =* 0.01) but showed no differences in scores on the MoCA (median 27 vs 26) or the MMSE (median 30). ACE-R scores were significantly lower for TIA subjects in the attention (median 16 vs 56, *P* < 0.01), memory (median 34 vs 60, *P* < 0.001), fluency (median 30 vs 39, *P* < 0.01), and language (median 26 vs 30, *P* = 0.03) subdomains of ACE-R, when compared to non-TIA subjects. For the other neuropsychological measures (Table 2), TIA subjects had lower scores in the memory (median 44 vs 52, *P <* 0.01) and processing speed (median 45 vs 62, *P <* 0.01) but not executive function, when compared to non-TIA subjects.

**Table 1:** Demographic, clinical, and cognitive screen data for PREVENT subjects.

Characteristics	TIA, n = 95	Non-TIA, n = 51	*P* values
**Age, years (**Mean±SD)	67.9 ± 9.4	64.9 ± 9.9	0.07
**Sex, female**	47%	67%	0.32
**DWI lesions (% of subjects)**	25 (26%)	N/A	NA
**Mean DWI lesion volume, mL (**±SD)	0.63 ± 0.87	N/A	N/A
**NART (Verbal IQ; Intellect)** (Mean±SD)	106.6 ± 9.6	109.6 ± 8.7	0.07
**FRS, n**	Low: 14 (14.7%)	Low: 25 (49.0%)	0.02
	Moderate: 20 (21.1%)	Moderate: 16 (31.4%)	
	High: 52 (54.7%)	High: 9 (17.6%)	
	N/A: 9 (9.5%)	N/A: 1 (2.0%)	
**ICV, mL (**Mean±SD)	1394.7 ± 123.5	1374.8 ± 122.7	0.36
**WM hyperintensities volume, mL** (Mean±SD)	11.4 ± 13.1	9.1 ± 10.9	0.28
**Stroke Etiology**		N/A	
Atrial Fibrillation	9 (9.5%)		
Cardioembolic	2 (2.1%)		
Cryptogenic	53 (55.8%)		
Hypotension	1 (1.1%)		
ICAD	1 (1.1%)		
Large Artery	6 (6.3%)		
Large Vessel	1 (1.1%)		
Migraine Aura	3 (3.2%)		
Migraine	3 (3.2%)		
Peripheral Vertigo	1 (1.1%)		
Small Vessel	15 (15.8%)		
**ACE-R**			
Median (IQR)	91 (86–96)	95 (91–97.8)	<0.01
**MoCA**			
Median (IQR)	26 (23–27)	27 (25–28.8)	0.29
**MMSE**			
Median (IQR)	30 (28–30)	30 (30–30)	0.64

IQR: interquartile range, 25^th^ and 75^th^ percentiles were presented; DWI: Diffusion-weighted imaging; NART: National Adult Reading Test; FRS: Framingham Risk Score (FRS); ICV: Intracranial volume; WM: White matter; ICAD: Intracranial Atherosclerotic Disease; ACE-R: Addenbrooke’s Cognitive Assessment-Revised; MoCA: Montreal Cognitive Assessment; MMSE: Mini-Mental State Examination.

**Table 2:** Descriptive statistics and Wilcoxon rank-sum test for cognitive domains and neuropsychological tests for PREVENT subjects.

		TIA, n = 95	Non-TIA, n = 51	*P* values
Domain	Neuropsychological Test	Median (IQR)	Median (IQR)	
**Memory**	BVMT-R Total	47 (37–55)	53 (42–63)	<0.01
	BVMT-R Delayed	53 (39–61)	59 (50–62)	0.02
	ACE-R Memory	49 (37–56)	56 (52–60)	<0.01
	WHO/UCLA AVLT-Total	53 (47–60)	59 (53–67)	<0.01
**Executive Function**	CLOX-1	57 (53–57)	57 (53–57)	0.49
	TMT-B	55 (45–63)	58 (47–63)	0.42
**Processing Speed**	WAIS-DS Coding	53 (47–60)	60 (50–63)	<0.01
	TMT-A	55 (47–63)	58 (50–63)	0.09

IQR: interquartile range, 25^th^ and 75^th^ percentiles were presented; BVMT-R: Brief Visuospatial Memory Test-Revised; ACE-R: Addenbrooke’s Cognitive Examination-Revised; WHO/UCLA-AVLT: WHO/UCLA Auditory Verbal Learning Test; CLOX-1: Clock drawing; TMT-A/B: Trails Making Test; WAIS DS Coding: Digit Symbol Coding.

### Association of TIA with FA and MD

After controlling for age, sex, premorbid intellect, FRS, ICV, WM hyperintensities volume, we observed that the relationship between TIA group and lower FA and higher MD values were related to the ROIs in the WM tracts. Specifically, compared with non-TIA subjects, TIA subjects had statistically significantly lower FA in the SLF (-0.29 units), UF (-0.26 units), and genu (-0.22 units). Also, compared with non-TIA subjects, TIA subjects had statistically significant higher MD in the fornix (0.36 units). Lower FA and higher MD were associated with age (*P <*0.01) and WM hyperintensities volume (*P <*0.01), and higher MD was associated with FRS (*P <*0.01). Table 3 shows the association of TIA within each ROI of the WM tract.

**Table 3:** Linear mixed-effects regression analysis for FA and MD controlling for age, sex, premorbid intellect, Framingham Risk Score (FRS), intracranial volume (ICV), WM hyperintensities volume, and the interaction of FA and MD with group (TIA subjects and non-TIA subjects).

	FA		MD	
	Estimate (95%CI)	*P*	Estimate (95% CI)	*P*
	TIA vs. Non-TIA		TIA vs. Non-TIA	
**CG**	-0.05 [-0.23–0.13]	0.578	0.06 [-0.05–0.17]	0.292
**PHC**	-0.07 [-0.24–0.11]	0.441	0.01 [-0.10–0.11]	0.936
**Fornix**	-0.16 [-0.34–0.01]	0.069	0.36 [0.26–0.47]	<0.001
**SLF**	-0.29 [-0.46- -0.11]	0.001	0.03 [-0.08–0.14]	0.598
**UF**	-0.26 [-0.43 - -0.08]	0.004	0.03 [-0.08–0.14]	0.603
**Genu**	-0.22 [-0.39- - 0.04]	0.016	0.04 [-0.07–0.15]	0.453

The coefficients reported were standardized. SE: standard error; FRS: Framingham Risk Score; ICV: Intracranial Volume; CG: Cingulum Gyrus; PHC: Parahippocampal Cingulum; SLF: Superior Longitudinal Fasciculus; UF: Uncinate Fasciculus.

### Association of DTI FA and MD with neuropsychological test scores

**Fig 2:**
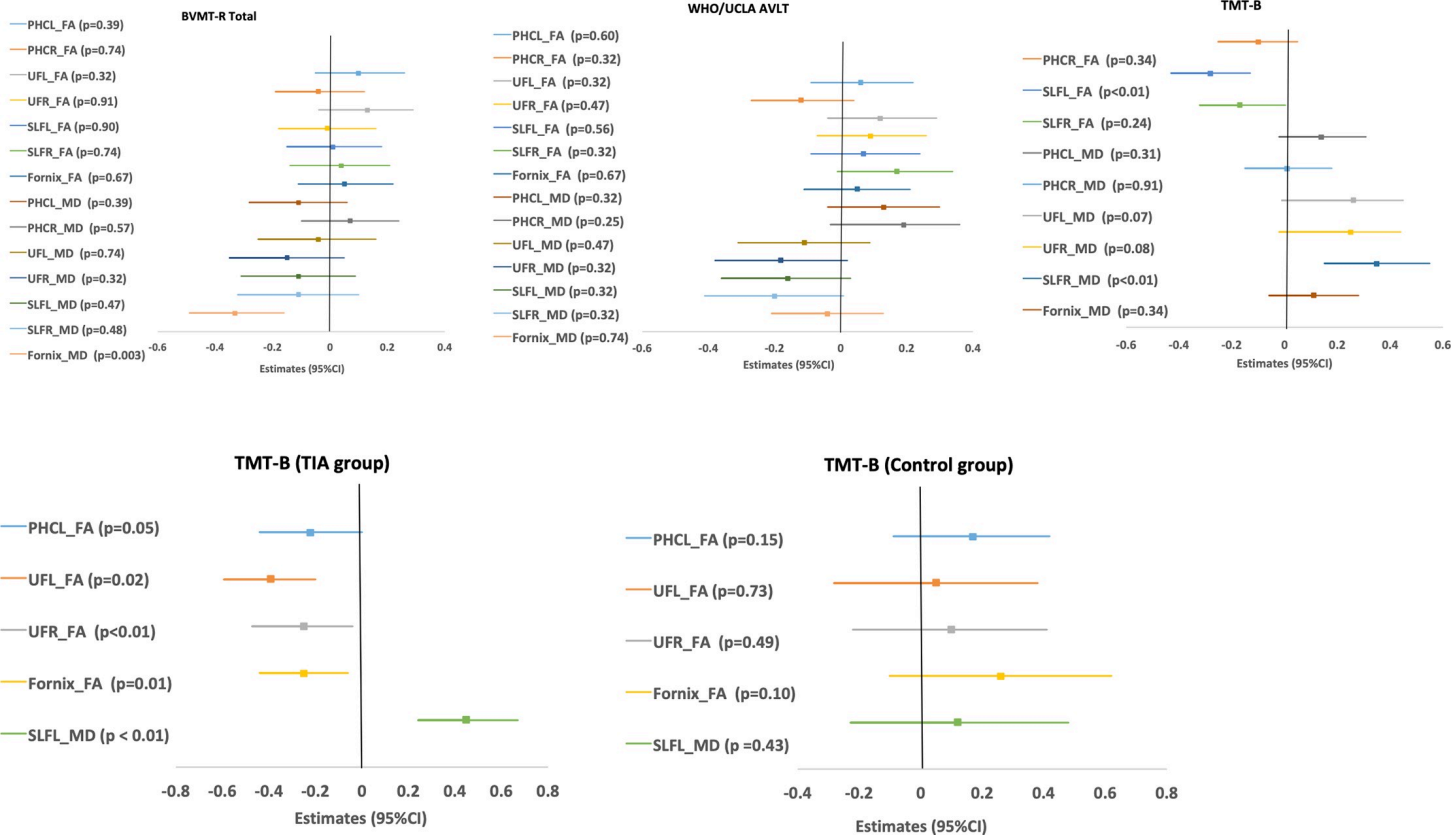
(A) Forest plots of the point estimates and 95% confidence interval (CI) for the relationship between DTI tracts and cognitive tests (TIA or non-TIA did not modify the relationships). Notes: The adjusted covariates include age, sex, premorbid intellect, group (TIA or non-TIA subjects), and WM hyperintensities volume. BVMT-R = Brief Visuospatial Memory Test-Revised; WHO/UCLA-AVLT: WHO/UCLA Auditory Verbal Learning Test; TMT-B: Trails Making Test. PHCL = parahippocampus, left; PHCR = parahippocampal cingulum, right; UFL = uncinate fasciculus, left; UFR = uncinate fasciculus, right; SLFL = superior longitudinal fasciculus, left; SLFR = superior longitudinal fasciculus, right. For BVMT-R total and WHO/UCLA AVLT related regressions. (B) Forest plots of the point estimates and 95% confidence interval (CI) for the relationship between DTI tracts and cognitive tests (TIA group modified the relationships). Notes: Results presented were based on multiple linear regressions where two-way interactions were significant statistically. The adjusted covariates including age, sex, premorbid intellect, group (TIA or non-TIA), and WM hyperintensities volume. TMT-B: Trails Making Test. PHCL = parahippocampus, left; UF = uncinate fasciculus, (L for left and R for right); SLFL = superior longitudinal fasciculus, left.

Fig 2A summarizes the relationships between neuropsychological test scores and DTI measures, after adjusting for age, sex, premorbid intellect, TIA or non-TIA, and WM hyperintensities volume. These differences were associated with DTI parameters of lower FA and higher MD in the fornix, SLF, and UF. Higher MD in the fornix was associated with lower BVMT-R scores (*P* = 0.003). Also, higher FA in the left SLF was associated with lower TMT-B time (indicating better performance on the test) (*P* < 0.01). Finally, higher MD in the right SLF was associated with higher score (indicating worse performance) on the TMT-B (*P* < 0.01).

Fig 2B shows the relationship between DTI WM tracts and neuropsychological tests that were modified by TIA. In TIA subjects lower FA in the left and right UF and fornix were related to longer timed performance on the TMT-B (the estimates < 0 and *P* = 0.02, *P*< 0.01, *P* = 0.01, respectively), and higher MD in the left SLF was related to longer timed performance on the TMT-B (the estimates > 0 and *P*< 0.01). None of these relationships were observed in the non-TIA subjects (all *P*>0.1). Results presented are based on multiple linear regressions and for the cognitive tests and DTI tracts where no two-way interactions were significant statistically.

## Discussion

We assessed the WM microstructure and cognition of TIA subjects compared to non-TIA subjects, as well as, the relationship of diffusion measures to cognitive outcomes. Consistent with our hypothesis, TIA subjects exhibited differences in DTI measures, corresponding to damaged microstructure. We found higher MD values in the fornix and lower FA values in the SLF, UF, and genu. DTI measures of FA and MD measured from the fornix, SLF, and UF predicted cognitive performance in both visual memory and executive function. Age, vascular risk factors (FRS), and WM hyperintensities volume were independently associated with higher MD values, and age and WM hyperintensities volume with lower FA values. We did not find a relationship between the presence of a DWI lesion and any of the DTI measurements.

In line with previous studies investigating cognition in TIA subjects [1], our study showed that PREVENT TIA subjects had worse cognitive outcomes involving multiple domains when compared to non-TIA subjects. Transient ischemic attack subjects showed lower scores on the ACE-R cognitive screen but no differences on the MoCA or the MMSE. However, it should be emphasized that objective differences in neuropsychological profiles are small (i.e., small effect size) and cognitive performance between PREVENT TIA subjects and non-TIA subjects cannot be discriminated by commonly used cognitive screening tests (MMSE or MoCA). Specifically, we observed cognitive differences for tests assessing memory, executive function, and processing speed with TIA subjects performing worse than non-TIA subjects. Lower cognitive scores were not associated with WM hyperintensities volume but were instead associated with higher age, FRS score, and premorbid intellect, highlighting the vascular burden of disease and the importance of intellect as a covariate in neuropsychological studies of dementia. The observed pattern of cognitive changes, with both memory (verbal and visual) and executive dysfunction, may support the presence of both vascular and AD pathology as contributors to the impairments [29].

Three candidate fasciculi are known to link components of memory networks: PHC, fornix, and the UF [30]. A study in healthy adults found age-related decline in recall to be associated with degradation of fornix microstructure, which is an important tract for episodic memory [30]. We extend these results to our study population, which found that fornix MD is predictive of performance on the BVMT Total Recall even after controlling for age, which points to incipient disease above what can be explained by age that contributes to WM degradation. Metzler-Baddeley and collaborators (2011), also found changes in UF microstructure linked to visual memory. We found no relationship with DTI parameters of other WM tracts for either BVMT or WHO/UCLA-AVLT. However, we measured widespread abnormalities in DTI parameters involving the right SLF that were associated with increased response time on TMT-B. The SLF is a bidirectional bundle that is necessary for processes such as attention, memory, and language [31]. Our study found that FA and MD as measured in the left and right SLF predicted performance in tests of executive function. As these results cannot be attributed to group status, it is implied that the integrity of the normal aging brain slowly worsens and, thus, deteriorates the connectivity required to effectively carry out these cognitive tasks. This study has shown that increased response times observed on the TMT-B were related to reduced FA in the left and right UF, and fornix, and with increased MD values in the left SLF that were only detected in TIA subjects. Thus, the results suggest that each of these tracts, which connect the frontotemporal regions, make critical but differential contributions to the mechanisms underlying cognitive changes and support distinct components of memory [30]. Also, the observed WM changes provide a plausible structural basis for selective loss of function in executive function through associations with non-fornix tracts, such as the SLF, and UF [32].

A few important limitations must be acknowledged. First, WM structures, such as the fornix, are surrounded by CSF and may be particularly prone to partial volume effects (PVE), which would result in lower FA and higher MD values. The use of fluid-attenuated inversion recovery (FLAIR) diffusion-weighted acquisition sequences may have reduced CSF contamination [33] but were not available for this study. Very small structures such as the cingulum may also be affected by PVE given that they are surrounded by grey matter. Since the DTI voxel size was identical for all subjects, effects of PVE are most likely systematic across groups although it is conceivable that TIA group-specific atrophy (i.e., TIA) in certain structures (i.e., fornix and cingulum) may be preferentially vulnerable to PVE over that of other groups and structures. Second, more complex high angular resolution diffusion imaging (HARDI) models may have given more resilience in light of multiple crossing fibre populations (notably the SLF) compared to the simpler but very well-established tensor model used here. Our study was exploratory, determining the relationship of WM structure and cognitive dysfunction. The strength of the relationship of structure with cognitive function have been reported in other aging studies, but the modification of these correlation with the TIA supports the detection of preclinical disease in a susceptible patient population that have been exclusively documented in neurodegenerative disease e.g. AD and FTD. Despite these limitations, our finding that underlying WM microstructure in multiple structures is predictive of cognitive functioning affords additional evidence of clinical utility for DTI metrics that will require confirmation in large cohorts of TIA subjects and with longitudinal analysis.

Transient ischemic attack subjects as identified by the PREVENT protocol might be an important patient group to study preclinical dementia [34, 35]. The presence of baseline cognitive changes in this high-risk TIA group and the results from exploring WM tracts as neuronal substrates of cognitive performance suggests that DTI measures may prove useful biomarkers during a prodromal stage of dementia [30, 36]. The research of Teipel and colleagues highlights an important temporal window as AD progresses that coincides with changes in DTI parameters beginning in the WM of limbic tracts and eventually affecting frontal-fiber pathways [37]. As such, utilizing biomarkers for both neurodegenerative and cerebrovascular pathology may improve predictive ability in clinical trials of dementia since etiology of cognitive changes is unknown. With no current treatments for dementia, control of modifiable vascular risk factors has been proposed as an important dementia-preventing alternative. Utilizing dementia as a primary outcome measure [4] in clinical trials investigating the efficacy of vascular risk-factor reduction treatments for prevention is problematic since significant neuronal and WM damage has occurred by the time cognitive impairment is detected [4]. Our results support the approach for determining the risk of cognitive decline in individuals presenting with TIA using WM deterioration as a surrogate of cognitive impairment. In the future, longitudinal data might help determine whether DTI parameters are a reliable and robust biomarker to determine the risk of cognitive decline and sensitive to preventative or disease modifying treatment [38].
